# Synergistic DES–Microwave Fractionation of Agri-Food Biomasses in a Zero-Waste Perspective

**DOI:** 10.3390/molecules30173588

**Published:** 2025-09-02

**Authors:** Luca Carlomaria Pariani, Franca Castiglione, Gianmarco Griffini, Letizia Anna Maria Rossato, Eleonora Ruffini, Alberto Strini, Davide Tessaro, Stefano Turri, Stefano Serra, Paola D’Arrigo

**Affiliations:** 1 Department of Chemistry, Materials and Chemical Engineering “Giulio Natta”, Politecnico di Milano, p.zza Leonardo da Vinci 32, 20133 Milano, Italy; lucacarlomaria.pariani@polimi.it (L.C.P.); franca.castiglione@polimi.it (F.C.); gianmarco.griffini@polimi.it (G.G.); letiziaanna.rossato@polimi.it (L.A.M.R.); eleonora.ruffini@polimi.it (E.R.); davide.tessaro@polimi.it (D.T.); stefano.turri@polimi.it (S.T.); 2 Istituto per le Tecnologie della Costruzione-Consiglio Nazionale delle Ricerche (ITC-CNR), via Lombardia 49, 20098 San Giuliano Milanese, Italy; alberto.strini@itc.cnr.it; 3 Istituto di Scienze e Tecnologie Chimiche “Giulio Natta”-Consiglio Nazionale delle Ricerche (SCITEC-CNR), via Luigi Mancinelli 7, 20131 Milano, Italy

**Keywords:** deep eutectic solvents, fractionation, microwaves, lignocellulose biomass, sustainability, ^13^C CP-MAS NMR, lignin, circular economy, agri-food waste

## Abstract

The growing demand for sustainable biorefinery approaches calls for efficient, environmentally benign strategies to valorize agricultural residues and ensure their complete utilization. This study explores the combination of deep eutectic solvents (DESs) and microwave heating technology as a greener process for the selective fractionation of agri-food waste residues in a zero-waste perspective. Within this framework, five representative biomasses were thoroughly investigated, namely brewer’s spent grain, raw and parboiled rice husks, rapeseed cakes, and hemp hurds. DES formulation was selected for its ability to solubilize and separate lignocellulosic components, enabling the recovery of a polysaccharide-rich fraction, lignin, and bioactive compounds. DES extraction was performed using both microwave heating and conventional batch heating, enabling a direct comparison of the two methods, the optimization of a more sustainable fractionation process, and the maximization of yields while preserving the functional integrity of the recovered fractions. A comprehensive characterization of the separated fractions was carried out, revealing that the two fractionation methods do not yield significant differences in the composition of the primary components. Moreover, a ^13^C CP-MAS NMR analysis of the recovered lignins demonstrates how this analytical technique is a real fingerprint for the biomass source. The results demonstrate the great potential of microwave DES-mediated fractionation as a mild, tunable, and sustainable alternative to conventional methods, aligning with green chemistry principles and opening new approaches for the full valorization of waste byproducts

## 1. Introduction

The ever-increasing generation of agri-food waste represents one of the major environmental challenges of our time, calling for urgent and sustainable valorization strategies aligned with circular economy principles. These residues, often rich in lignocellulosic biomass, are generally underutilized and are frequently disposed of by incineration or landfilling, resulting in significant environmental burdens. In a zero-waste context, such biomasses should instead be regarded as valuable feedstocks for the production of renewable materials and chemicals [1,2]. In this context, five important representative agri-food residues were selected for fractionation, namely brewer’s spent grain (BSG), raw and parboiled rice husks (r- and p-RHs), hemp residues, and rapeseed cakes. These materials are generated at large volumes annually and are typically underexploited despite their high content of recyclable components, such as carbohydrates, cellulose, proteins or triglycerides.

BSG is the most abundant byproduct of brewing, accounting for approximately 85% of the total waste generated: producing 100 L of beer yields around 20 kg of discarded wet BSG, or roughly 14 kg when dried. This biomass is rich in proteins, fibers, and lignin. Its high moisture content (~75%) is responsible for its perishability, which often limits its use to animal feed or composting, requiring its prompt removal from the brewery. Typically, it is repurposed locally as cattle feed or agricultural fertilizer. However, this solution becomes impractical when breweries are located in densely populated urban areas or in remote, non-agricultural regions (e.g., mountainous areas), where rapid redistribution is unfeasible. In such cases, BSG management becomes both economically burdensome and environmentally inefficient, resulting in the loss of a potentially valuable resource.

Rice husks (RHs) are a silica-rich byproduct of rice milling and constitute a significant waste stream in rice-producing countries. Their structural rigidity and high ash content make them challenging to process, yet they hold considerable potential as a renewable resource. RHs are recovered from rice (*Oryza sativa*), which represents one of the world’s most essential staple crops, with global rough rice (also known as paddy rice) production exceeding 800 million tons in 2024. Its critical role in global nutrition is underscored by the fact that it serves as a primary food source for nearly three billion people worldwide. Rough rice consists of an outer husk and the brown rice kernel, which are separated during the dehusking process. RHs, obtained as a byproduct from this stage, are a major form of agricultural waste and rank among the most abundant residues in the rice industry. As husks account for approximately 20–25% of the total paddy rice mass, annual global rice husk generation is estimated to exceed 160 million tons. Currently, this biomass is primarily utilized for energy production. Parboiled rice, processed through a hydrothermal treatment involving hydration, starch gelatinization, and drying, has gained increasing market relevance due to its enhanced nutritional profile and improved physical properties. It typically commands two to three times the economic value of white rice. At present, around 20% of global rice undergoes parboiling. As this process is commonly applied to rough rice, the resulting parboiled rice husk (p-RH) represents a growing proportion of the total rice husk output, with projections indicating a continued rise in the coming years.

Hemp hurds, obtained from the industrial processing of hemp for stalks and seeds, have a high cellulose content and relatively low lignin content, making them attractive for biomass valorization. They are the residual material obtained after fiber separation and have only minor applications, such as in compost and adsorbent bedding. Similarly, rapeseed cakes, a byproduct of rapeseed oil refining, are typically left on the field or burnt, despite their considerable lignocellulosic fraction.

Rapeseed is a major oilseed crop in Europe with France, Germany, Poland, and the United Kingdom as the leading producers. Rapeseed press cakes are the residual material left after defatting rapeseed by mechanical extraction methods, such as screw pressing. These cakes contain approximately 10–18% of oil, which could be submitted to an additional solvent extraction step.

In this context, the valorization of these five feedstocks is of great interest and particularly relevant in countries where there is a great production of these wastes. Utilizing such agricultural residues as secondary raw materials could represent a more sustainable alternative to their combustion for energy in a circular economy perspective [3,4,5].

Deep eutectic solvents (DESs) have gained attention among emerging technologies, as they are formulated with a wide variety of components, often extracted or obtained from renewable sources [6,7,8,9,10]. As such they are particularly suitable for sustainable processing as they offer highly valuable characteristics, including low toxicity, high recyclability, and tunable solvating properties [11,12,13,14,15,16]. Even more promising is the use of reactive deep eutectic solvents (RDESs), which can simultaneously serve as separation media and reaction media in lignin valorization processes [17,18,19,20]. All these characteristics make DESs highly suitable for biomass fractionation processes, particularly in the treatment of lignocellulosic agri-food wastes [21]. The authors have recently set-up two different multistep fractionation processes mediated by deep eutectic solvents for BSG and rice husk treatment, which provided a full recovery of the different components of the waste biomass [22,23]. These processes were based on a conventional heating methodology (CHM). In this mainframe, a substantial improvement of the processes has been investigated by studying the potential use of microwave technology (MWT) [24]. In recent times, in fact, the use of microwave power in chemistry has seen a notable expansion of interest, with applications in the synthetic (organic [25,26], inorganic [27] and materials science [28,29,30]), extractive [31], and analytical [32] fields both at laboratory- and industrial-scale [33,34]. The main effect of the microwaves application is the selective heating of the mass in the process, with several advantages compared to traditional contact methods, including the speed and uniformity of the heating action [35]. The possible presence of specific mechanisms other than heating has also been debated for several years [36,37,38], but there is no conclusive evidence available on this subject. However, there are many indications of the direct advantages compared to traditional heating methods, including the reduction in secondary products formation, the enhancement of yields, the reduction in treatment times, and the general increase in the energy efficiency of the processes, which make the application of microwaves extremely interesting in the field of sustainable chemistry [39].

Based on these premises, in the present work, the authors have performed a deep comparison between the already stated conventional heating mode (CHM) and a novel microwave-assisted DES-based fractionation strategy with the final aim being to set up a more sustainable complete treatment of relevant biomasses. The current study expands the previous approach, with particular attention paid to comparing the final yields, as well as the composition and characteristics of polysaccharide and lignin fractions. This approach, leading to a nearly complete breakdown of the rigid lignocellulosic matrix, is designed to align with zero-waste objectives by enabling the recovery and reuse of all major biomass fractions. The study focuses on optimizing the fractionation process, characterizing the separated components, and evaluating their potential applications in sustainable material development. By integrating green chemistry tools, such as DESs and microwave technology, this work offers a scalable, environmentally benign route for efficiently transforming biomass waste into valuable bio-based products.

## 2. Results and Discussion

In this study, five abundant waste biomasses from agri-food production were selected (see Section 3.1 for details). The aim was to establish a sustainable fractionation process and to conduct an in-depth investigation of the resulting fractions, with particular emphasis on the lignin- and polysaccharide-enriched fractions. Complete separation and characterization of all components of these complex lignocellulosic biomasses are mandatory for the implementation of any valorization strategy.

**Figure 1:**
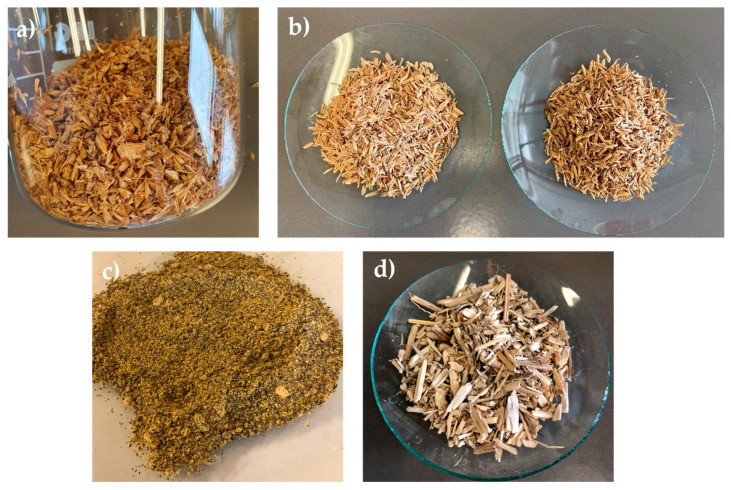
Pictures of fractionated biomasses. (**a**) Brewer’s spent grain; (**b**) Rice Husks (raw on the left and parboiled on the right); (**c**) rapeseed cake (powdered); (**d**) hemp hurds.

Samples of the starting biomasses employed in the present work are illustrated in Figure 1. As soon as they were received, all biomasses underwent a first drying treatment to ensure proper conservation of the materials and prevent microbial degradation, which can occur under high-moisture conditions. The water content of the biomasses accounted for 4–5% for BSG and RHs, for 2% for hemp hurds (from now named Hemp), for 9% for rapeseed cake (from now named Rapeseed).

### 2.1. Biomasses Composition Analysis

#### 2.1.1. Composition

The present examined lignocellulosic biomasses constitute an intricate material where all components are closely related to each other. To separate and quantify the main constituents, namely soluble carbohydrates, lipids, hemicellulose, cellulose, inorganic material, and lignin, a multistep procedure was performed based on a classical fractionation method involving successive water and acid treatments (see Section 3.3 for details). The composition of the studied biomasses is reported in Table 1.

In Rapeseed, lipids account for 15 wt.% of the starting biomass. Proteins were determined using a standard alkaline method with NaOH 0.5 M at r.t. for 1 h, giving a protein content of 36 wt.% in the starting material.

#### 2.1.2. Hemicellulose Fraction Analysis

The complete composition of the isolated hemicellulose fraction, expressed in terms of monosaccharide components, was determined by GC-MS analysis using a previously reported method [22] which takes advantage of a three-step procedure including total hydrolysis, reduction and acetylation. The results are reported in Table 2 and show that in all biomasses the main components are arabinose and xylose, with a small amount of galactose. Traces of rhamnose were detected in BSG and RHs, whereas mannose was present in rapeseed and hemp. The detected glucose likely results from cellulose hydrolysis during the hemicellulose dissolution.

### 2.2. Multistep Microwave Assisted Fractionation Process

**Figure 2:**
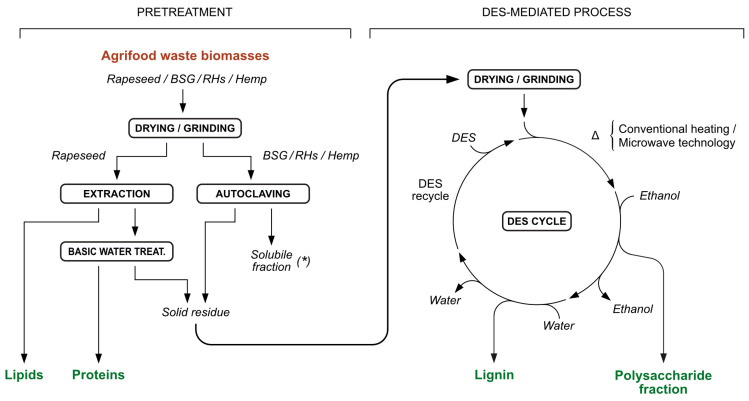
Complete DES-mediated fractionation multistep process for the waste biomasses. Soluble fraction (*) contains monosaccharides, β-glucans, and hemicelluloses.

The cascade multistep fractionation process, as illustrated in Figure 2, was thoroughly investigated and is described in detail herein. It consisted of two main steps: Firstly, the biomasses underwent a pretreatment, enabling the separation of the water-soluble components (BSG, RHs, and Hemp), or in the case of Rapeseed, lipids by a solvent-mediated extraction; the second step, referred to as the DES-mediated process, involved solubilizing the solid residues from the first step in DES under heating (CHM or MWT). This was followed by two selective precipitation steps to recover a polysaccharide fraction and a lignin fraction, with the final stage involving DES recycling.

#### 2.2.1. Water Thermal Process

The first pretreatment involved a hydrothermal process in order to separate the portion of the biomasses that are soluble in hot water (see Figure 2). This portion mainly contains soluble sugars, amino acids, and proteins which can be further exploited. In the case of BSG, the authors demonstrated in a previous work that this fraction, mainly composed by monosaccharides, β-glucans, and hemicelluloses, was relevant in terms of quantity because it accounted for about 25 wt.% of the starting material, and had already been successfully employed as an effective growth medium for different microbial fermentations. The basidiomycetous yeast *Phaffia rhodozyma*, the oleaginous yeast *Yarrowia lipolytica*, some PUFA-producing fungi and selected bacterial strains from the genus *Rhodococcus* and *Streptomyces* were successfully cultivated on BSG medium [22,40]. In the case of RHs, the amount of the soluble fraction did not exceed 2% of the starting biomass, and the exploitation of that fraction was performed by solvent extraction for the recovery of interesting phenylpropanoids and fatty acids. Also, for Hemp, this first treatment in water did not provide valuable products, as only a small percentage of the biomass was dissolved. However, rapeseed has been treated with basic water, because these materials have been shown to include a fair quantity of proteins in their backbones that can be separated in this way (see Section 3.4).

#### 2.2.2. DES-Mediated Process

**Figure 3:**
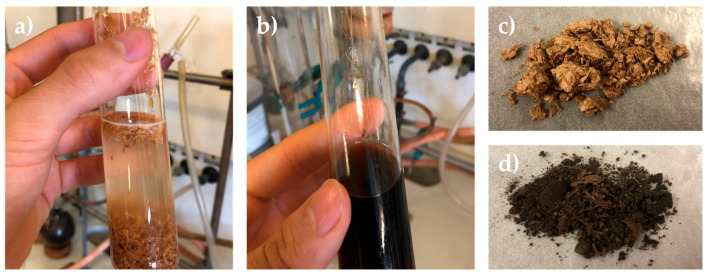
Different steps of biomass fractionation (reported here for brewer’s spent grain): (**a**) suspension of the biomass in DESs; (**b**) solubilization of biomass in a DES-mediated process; (**c**) recovered polysaccharide fraction; (**d**) recovered lignin fraction.

The DES-mediated fractionation process was carried out for all biomasses under both CHM and MWT conditions to compare the two heating methods. The biomasses were suspended in a DES solution composed of choline chloride/l-lactic acid (1:5 molar ratio) (see Figure 3a).

The biomasses were not soluble at r.t. but, as previously demonstrated, complete solubilization occurred after 24 h of CHM at 130 °C under magnetic stirring (see Figure 3b) [22]. When combined with DESs, microwave irradiation was expected to accelerate lignocellulosic deconstruction, enabling more efficient separation of biomass components, such as cellulose, lignin, and hemicellulose [41]. Preliminary experiments were conducted to identify optimal conditions for microwave-assisted fractionation in order to reach the highest extraction efficiency. Treatments were carried out at 2.45 GHz with an applied power of 300 W. Following the application of microwaves, the temperature rose from ambient to 130 °C in approximately 5 min and was then maintained for varying durations. Treatments shorter than 120 min left a substantial insoluble residue still suspended in the DESs. Quantitative solubilization, evidenced by a clear solution free of suspended solids, was achieved only after 360 min of irradiation at 130 °C, highlighting the need for prolonged exposure to ensure complete dissolution of the biomass matrix. Figure S6 shows the optimization of the microwave residence time using rapeseed as a reference biomass. The method was developed by analyzing the insoluble residues of biomass over time, keeping the set-point parameter as the obtained insoluble residue observed from the standard 24 h batch fractionation. For the correct comparison between microwave and batch reactor, 6 h was used as the optimal operative condition. Then, the identified optimal conditions included a DES composed of choline chloride (ChCl):l-lactic acid (1:5), microwave power of ~300 W, a temperature of 130 °C, and a total irradiation time of 6 h. This treatment time is in stark contrast to the traditional process, which required 24 h at 130 °C for complete dissolution. It is known that microwave heating of materials may induce structural modifications in lignocellulosic biomass, leading to a better final extraction yield for all components. Its main effect is due to microwave interaction with molecular dipoles, such as water molecules or hydroxyl groups (–OH). Moreover, the presence of highly polar DES molecules as the solvent results in strong microwave absorption throughout the entire sample mass. This results in volumetric heating, which is both rapid and uniform, in contrast to the surface-based heating typical of conventional thermal methods. As a consequence, the disruption of intermolecular interactions—such as hydrogen bonds and van der Waals forces—in our case within lignin, cellulose, and hemicellulose, has been significantly accelerated, allowing full skeleton destructuration with a resulting reduction in cellulose crystallinity and the promotion of lignin recovery, thereby enhancing biomass solubilization and extraction efficiency.

**Figure 4:**
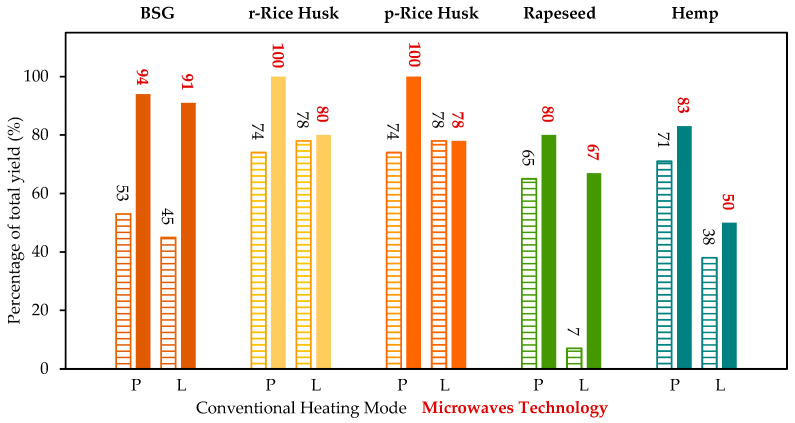
Recovery yields (mass of recovered fraction/mass of component in biomass × 100) of the polysaccharide fraction (P) and lignin-enriched fraction (L) obtained with the conventional heating mode (CHM, dashed bars, black numerical values) and with microwave technology (MWT, plain bars, red numerical values). Estimated standard deviation ± 1% (measured laboratory value for this technique).

Figure 4 illustrates the yields of the polysaccharide and lignin fractions, comparing the results obtained using the conventional thermal mode (dashed bars) with those achieved through microwave-assisted processing (solid bars). The data clearly demonstrate that microwave irradiation significantly enhances the extraction efficiency for both fractions across all investigated biomass types. Notably, BSG exhibits a two-fold increase in yield for both fractions, while rapeseed shows a ten-fold enhancement in lignin recovery, highlighting the pronounced effect of microwave-assisted extraction on process performance. Also, the comparison between the CHM and MWT demonstrates that microwave technology could greatly enhance the final yields of the fractions, leading to nearly 0% remaining of final unfractionated residue.

The polysaccharide fractions have been characterized in terms of their sugar composition. They have been completely hydrolyzed and analyzed through GC/MS analysis, as described in Section 3.3.1. The data reported in Table 3 show that these fractions are richer in cellulose, except for the fraction recovered from rapeseed, thus confirming the presence of a low quantity of cellulose in the starting biomass.

Furthermore, it is noticeable that the ratio of cellulose to hemicellulose significantly decreases when CHM technology is replaced with the MWT method. This observation suggests that microwave heating does not hinder the separation of cellulose but rather that it enhances the extraction efficiency of hemicellulose. Overall, both the hemicellulose/cellulose ratio (as shown in Table 3) and the polysaccharide recovery yield (illustrated in Figure 4) increase substantially from the CHM method to the MWT method.

**Figure 5:**
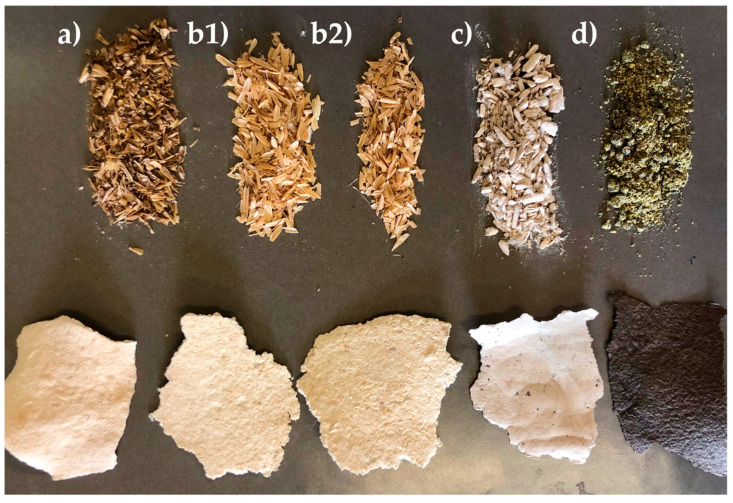
Paper specimens created from the MWT-cellulose-enriched fractions from the different biomasses ((**a**) BSG, (**b1**) r-Rice Husk, (**b2**) p-Rice Husk, (**c**) Hemp, and (**d**) Rapeseed).

A potential application of these fractions could be their exploitation in paper production. In fact, they have been used as starting material for demonstrative paper making at a laboratory scale using 5 g of the polysaccharide fraction. After the drying step, the samples were submitted to a standard bleaching process: they were treated with 10% H_2_O_2_ for 3 h under stirring at r.t. in order to obtain a substantial bleaching of the sheet of paper. The Rapeseed sample was the only sample not to clear up. Then, the samples were filtered and pressed in a mold for 24 h. The resulting materials are reported in Figure 5.

### 2.3. Lignin Analysis

The recovered lignin fractions were thoroughly characterized in terms of chemical, physical, thermal, and structural properties to provide essential information for developing further valorization strategies [42]. In fact, lignin possesses remarkable potential for novel applications, particularly in the creation of advanced materials, and its suitability for such uses depends strongly on its composition and intrinsic properties [43]. Moreover, lignin could be introduced into the DES system as a renewable, internally sourced hydrogen bond donor, thereby paving the way towards a potential “closed-loop” biorefinery process [44,45].

#### 2.3.1. GPC Results—Molar Mass Determination

Lignin samples extracted from the same biomass types by either the CHM or MWT exhibited similar chromatograms (reported in Figure S3) and comparable values of Mn, Mw, and Đ (reported in Table 4). These results indicate that the two methods employed for the fractionation yield lignins with essentially equivalent macromolecular properties.

#### 2.3.2. Total Phenolic Content

The total phenolic content of all recovered lignins was determined using a modified Folin–Ciocalteu (FC) assay (see Section 3.6.1 for details). For this analysis, samples were initially fully solubilized in DMSO before being incubated with the specific redox reagent (FC reagent). The phenolic content results are summarized in Table 5 as the vanillin equivalents (mmol/g of dry lignin), using the data from Protobind 1000 as the reference.

All five lignins obtained by MWT showed a 14–30% increase in vanillin equivalent content compared to those recovered by the CHM, as reported in Table 5. This enhancement is significant for future lignin valorization, as a higher phenolic hydroxyl content is crucial for potential applications in material science [43,46].

#### 2.3.3. Fourier Transform Infrared Spectroscopy

**Figure 6:**
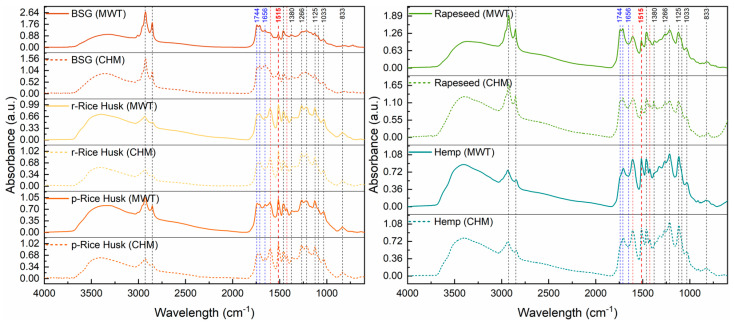
FTIR spectra of lignin extracted from (**left**) Brewers’ spent grain (BSG), raw Rice Husks (r-Rice Husk), and parboiled Rice Husks (p-Rice Husk), as well as from (**right**) Rapeseed and Hemp via either the conventional heating mode (CHM) or microwave treatment (MWT).

FTIR spectroscopy was applied to further assess the chemical composition of the recovered lignins. The absorption spectra and corresponding signal assignments are reported in Figure 6 and Table 6. No substantial differences were observed among lignins from Rice Husks, Rapeseed, and Hemp. However, lignin from BSG displayed a slightly different IR spectrum compared to the other samples. In particular, the peaks associated with the C=O stretching of carbonyl/carboxyl groups in carbohydrate species (i.e., at 1744, 1712, and 1656 cm^−1^) are much more intense than the characteristic signals at 1600 and 1424 cm^−1^ assigned to lignin aromatic skeletal vibrations, which are slightly visible in this case. This suggests the presence of residual cellulose/hemicellulose, likely due to the inherent chemical heterogeneity of this agri-food waste, which makes it difficult to extract extremely high-purity fractions.

#### 2.3.4. Thermal Behavior

**Figure 7:**
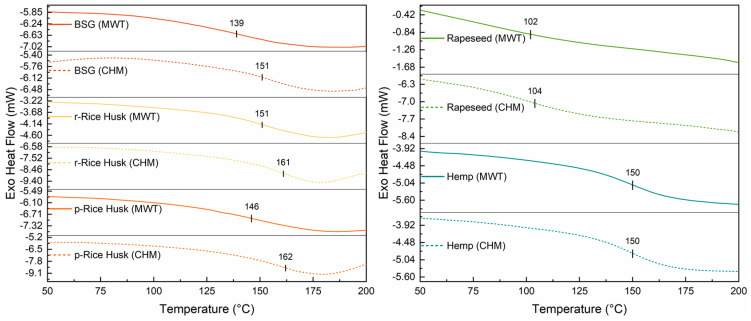
DSC second heating run of lignin extracted from (**left**) brewers’ spent grain (BSG), raw Rice Husks (r-Rice Husk), and parboiled Rice Husks (p-Rice Husk), as well as from (**right**) Rapeseed and Hemp lignocellulosic biomasses via either the conventional heating mode (CHM) or microwave (MWT) treatment. Estimated standard errors ± 3 °C.

Differential scanning calorimetry (DSC) was used to determine the thermal transitions of the extracted lignins. As shown in Figure 7 (left and right), BSG, RHs, and Hemp lignins displayed glass transition temperature (T_g_) values in the 139–162 °C range, with comparatively lower T_g_ values observed upon MW processing (see Table 7).

In contrast, rapeseed lignin had a slightly lower T_g_ compared with the other systems, correlating with its lower M_n_ (see Table 4). Interestingly, this type of extracted lignin also exhibits a larger abundance of phenolic groups (see the FC assay in Table 5), which may suggest the presence of a macromolecular network characterized by shorter chains of comparatively higher chemical (–OH) functionality.

Lignin from the same type of biomass (i.e., Rapeseed and Hemp) present comparable values of T_g_, M_n_, M_w_, and *Đ*. This indicates that the two methods employed for the fractionation yield lignins that are equivalent in terms of their thermal and macromolecular properties. Furthermore, the fraction extracted from rapeseed is characterized by a significantly lower glass transition temperature and average molecular weight compared to that recovered from hemp. This could stem from a difference in the content of functional groups between the two samples.

As for the fractions derived from r- and p-Rice Husks, it can be observed that the latter shows lower values of M_n_, M_w_, *Đ*, and T_g_ compared to the former. This is likely due to the parboiling treatment, a hydrothermal process that promotes the partial depolymerization of all the components of the lignocellulosic biomass, ultimately leading to reduced molecular weight and increased mobility of lignin macromolecular chains.

Moreover, when T_g_ values were plotted against 1/M_n_ (as shown in Figure S7), for both the CHM and MWT series, T_g_ was found to increase almost linearly with the value of M_n_, in accordance with the Flory–Fox equation [47].

#### 2.3.5. ^13^C CP-MAS NMR

Solid-state ^13^C cross-polarization magic-angle spinning (CP-MAS) NMR spectroscopy is a powerful and versatile technique to obtain a unique and detailed “fingerprint” of each lignin sample [23]. This is particularly valuable because lignin is a complex aromatic polymer, whose heterogeneous structure makes its characterization difficult. However, variations in the botanical source, extraction process (solvents), or subsequent treatments can induce changes in the structure and morphology of lignin that can be observed in solid-state NMR spectra [48]. Furthermore, solid-state NMR allows the direct analysis of lignin in its native state, preserving its intrinsic structural features and allowing the observation of the mobility and degree of crystallinity of the different regions [49].

**Figure 8:**
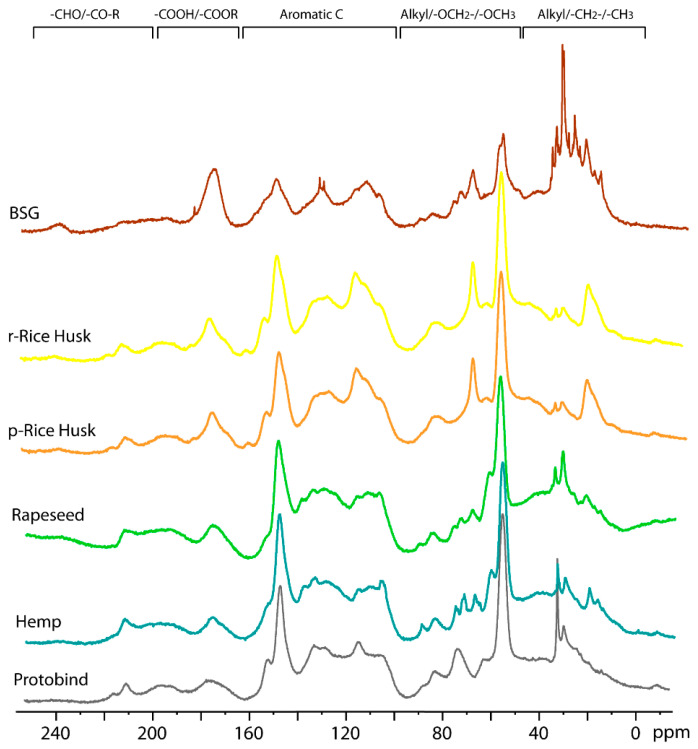
^13^C CP-MAS NMR spectra of all lignin extracts and Protobind lignin (grey line).

The effect of different types of treatment (conventional heating mode vs. microwave technology) on the structure and morphology of lignin was studied using ^13^C CP-MAS NMR on r- and p-Rice Husk extracted lignin samples (spectra reported in Figure S4). In both cases, the ^13^C CP-MAS spectra show a similar pattern, showing that no significant transformations or alterations occurred during the microwave treatment compared to the conventional heating mode. These data confirm the results already described in the previous sections. In the following Figure 8, the NMR spectra of the presently extracted lignin samples, obtained from different bio-sources and treated with the microwave-based procedure, are compared. Figure 8 shows the ^13^C CP-MAS spectra of the extracted lignin samples (BSG, r-Rice Husk, p-Rice Husk, Rapeseed, and Hemp) and the Protobind commercial lignin (grey line) for comparison.

**Figure 9:**
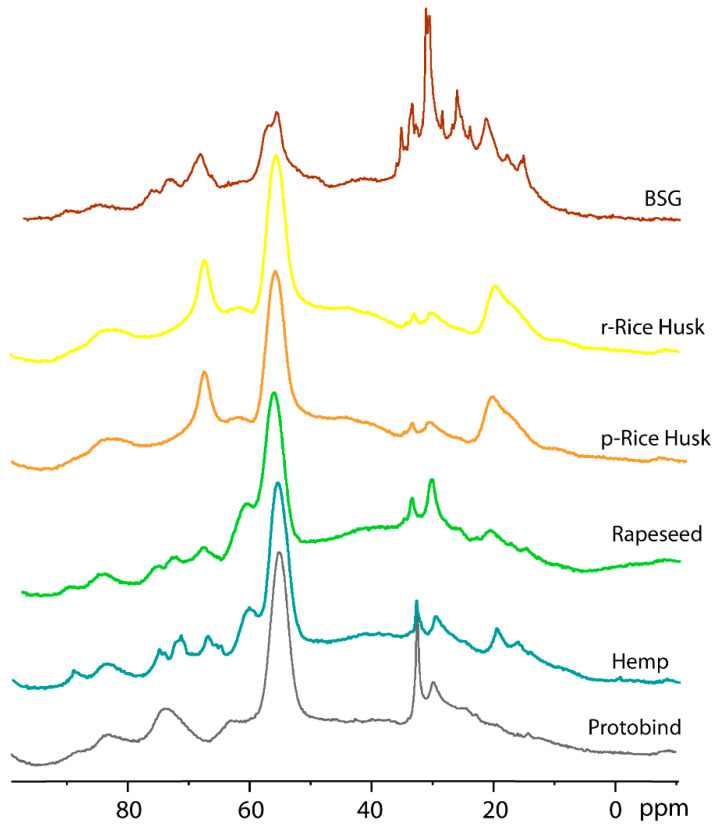
^13^C CP-MAS NMR spectra of all lignin extracts and the Protobind lignin (expanded region 100–0 ppm).

Five different spectral regions can be observed, as follows: (*1*) the 240–190 ppm region corresponding to aldehydes carbonyl groups; (*2*) the 90–160 range of carboxylic and ester groups; (*3*) the aromatic region at 160–100 ppm; (*4*) alkyl-O-CH_2_- and methoxy carbons at 95–45 ppm; (*5*) alkyl carbons in the 45–0 ppm. Similar to the Protobind lignin, a single peak is observed for all lignin extracts, with a chemical shift of 212 ppm for Hemp, Rapeseed, r- and p-Rice Husks samples, while the resonance shifts to 237 ppm for BSG lignin. The peak at 175 ppm due to carboxylic and ester groups has similar intensity for the Protobind, hemp, and rapeseed lignin, while its intensity increases for r-Rice Husk, p-Rice Husk, and BSG samples, thus indicating their higher amount of these groups. Another intense peak is observed at 148 ppm due to carbons on guaiacyl phenolic units and syringyl units [50]. This peak shows lower intensity for the BSG lignin extract compared to the other samples. The aromatic region shows a similar profile for Protobind, Hemp, and Rapeseed; r- and p-Rice Husks also have a similar profile to each other. However, the most significant differences are observed in spectral regions *4* and *5* (100–0 pp), which are expanded in Figure 9.

In spectral region *4*, the Protobind lignin shows two peaks at 74.5 and 63.7 ppm due to C-OH in the β-O-4 structural moiety with the –OH group at the α-position. The peak at 74.5 shows a different multiplicity in Hemp, Rapeseed, and BSG, while it is absent in r- and p-Rice Husks. Moreover, the peak at 63.7 ppm is shifted upfield at 60.6 ppm in hemp and rapeseed and downfield at 67 ppm in r-Rice Husk, p-Rice Husk, and BSG. The strong characteristic peak at 55.3 ppm due to methoxy carbons has a lower intensity for the BSG compared to the other extracts. Spectral region *5* is the one that most highlights the differences between the various samples and can, therefore, be considered the fingerprint of each sample. In particular, the peak at 19.5 ppm (methyl groups) has a higher intensity in r- and p-Rice Husk samples. Finally, it is interesting to note that the ^13^C CP-MAS spectrum of the BSG lignin extract shows an overall different profile compared to the other spectra with some sharp lines due to a certain degree of crystallinity of the sample.

## 3. Materials and Methods

BSG samples were provided by the “L’Orso Verde” Brewery (Busto Arsizio, Italy). The rice husk samples (from *Oriza sativa*) were kindly provided by Riso Scotti S. p. A. (Pavia, Italy) as raw Rice Husk (r-RH) derived from the processing of *japonica* (90%) and *indica* (10%) rice varieties and parboiled Rice Husk (p-RH) were derived from *indica* (75%) and *japonica* (25%) varieties. Hemp hurds from North Italian cultivations were kindly provided by Dr. Gianluca Ottolina from SCITEC-CNR (MI), and rapeseed cakes were provided from Poland by Prof. Dr. Aleksandra Grudniewska (Wroclaw University of Environmental and Life Sciences, Poland). Protobind 1000 (a mixed wheat straw/sarkanda grass lignin from the soda pulping of non-woody biomass) was purchased from Tanovis (Alpnach, Switzerland).

All air- and moisture-sensitive reactions were carried out using dry solvents and under a static atmosphere of nitrogen. Choline chloride (cat. C0329) and l-lactic acid (cat. L0165), d-(+)-galactose, and l-(−)-fucose were purchased from TCI (Milano, Italy), whereas l-(+)-rhamnose, l-(+)-arabinose, d-(+)-xylose, d-(+)-mannose, d-(+)-glucose, and the other reagents and employed solvents, used without further purification, were obtained from Merck (Merck Life Science S.R.L., Milan, Italy).

Thin-layer chromatography (TLC) Merck silica gel 60 F_254_ plates (Merck Millipore, Milan, Italy) were used for the analytical TLC.

### 3.1. Biomass Drying

As soon as they were received, all biomasses underwent a first drying treatment in a ventilated oven (60 °C for 24 h); then, they were finely ground with an electric grinder. The water content in the pristine biomasses was 4–5% for BSG and RHs, 2% for hemp hurds, and 9% for rapeseed cakes.

### 3.2. Preparation of DESs

The DESs were prepared by mixing choline chloride, the anhydrous hydrogen bond acceptor (HBA), with lactic acid, which constitutes the hydrogen bond donor (HBD), in the molar ratio 1:5. The mixture was stirred in a closed flask at 120 °C for 4 h until the liquid phase appeared completely homogeneous and clear. The product was then dried under vacuum and stored at room temperature in a desiccator in the presence of anhydrous calcium chloride until further use. The ^1^H NMR spectrum of the DES (choline chloride/l-lactic acid 1:5) employed for the full fractionation is reported in Supplementary Materials Figure S1.

### 3.3. Biomasses Treatment with DES

#### 3.3.1. Conventional Batch DES-Mediated Lignocellulose Process

Biomasses (25 g) were suspended in DES choline chloride/l-lactic acid (1:5) (250 mL) at 130 °C in a round-bottomed flask under magnetic stirring for 24 h. After cooling, ethanol (500 mL) was added gradually over 2 h in order to precipitate the polysaccharide fraction. The pellet was separated by centrifugation and filtration, washed repeatedly with ethanol and dried to give a final polysaccharide solid fraction (4–6 g) with a yield of 17–25% (*w*/*w* initial biomass). The filtrate was then concentrated by rotary evaporation under vacuum to eliminate ethanol. Water (500 mL) was then added, and the suspension was stirred for 24 h at 4 °C. The obtained precipitate was then centrifuged, filtered, and washed three times for 1 h with a solution of water/ethanol (9:1). After centrifugation, filtration, and solvent evaporation, the final fraction (B-lignin, 2.4 g) was recovered with a final yield of 10% (*w*/*w* initial biomass).

#### 3.3.2. Microwave DES-Mediated Lignocellulose Process

The biomasses were fractionated using an Ethos X microwave equipped with a fastEX-12 rotor (FKV, Milestone, Sorisole, Italy). Each biomass (20 g) was subdivided into five tubes, with 4 g of material accurately weighed into each tube. Subsequently, 40 mL of DES of choline chloride/l-lactic acid (1:5) was added to each tube (ratio biomass/DES of 1:10) and heated to 130 °C under magnetic stirring using a programmed temperature ramp. Specifically, the temperature was increased from r.t. to 130 °C over a period of 5 min and maintained at 130 °C for 6 h. The tubes were then cooled to r.t. and ethanol was added gradually over 2 h in order to precipitate the cellulose-enriched fraction. After centrifugation and filtration, the recovered residue was washed with ethanol and then dried to obtain a final solid cellulose-enriched fraction. The filtrate was then concentrated under vacuum. Water (400–500 mL) was then added and the suspension was stirred for 24 h at 4 °C. The obtained precipitate that was recovered after centrifugation and filtration was then washed with a solution of water/ethanol (9:1). The final lignin fraction was recovered by filtration and then dried. The final yields are reported in Figure 4.

### 3.4. Protein Extraction for Rapeseed Cakes

The proteins were extracted using the classical alkaline treatment (ASAP treatment). Briefly, 4 g of biomass was suspended in 200 mL of NaOH 0.5 M at r.t. for 1 h. The suspension was then filtered and acidified with HCl 2 M to pH 1. The solid proteic residue was then filtered and weighed. The final yield was 35%.

### 3.5. Determination of Biomasses Composition

The biomass compositions were determined with a known multistep procedure with minor modifications [51]. The raw biomass (1 g, reported as value a) was suspended and stirred in deionized water (150 mL) at 100 °C for 1 h. Then, after filtration, the solid was washed with deionized water (300 mL), dried in an oven at 80 °C, and weighed (reported as value b). The solid was treated with 1 N of H_2_SO_4_ (150 mL) at 100 °C for 1 h. Then, the suspension was filtered again, washed with water, and the solid was dried and weighed (value c). The solid was mixed at r.t. with 72% H_2_SO_4_ (10 mL) for 4 h and treated with 1 N of H_2_SO_4_ (150 mL) for 1 h under a reflux. After cooling, the solid was filtrated, dried, and weighed (reported as value d). The final residue was then calcinated in an oven at 600 °C for 6 h, and the residue was quantified (value e). The fractions of the different components were quantified with the following equations:

hemicellulose (%) = 100 × [(b − c)/a]

cellulose (%) = 100 × [(c − d)/a]

lignin (%) = 100 × [(d − e)/a]

#### Gas Chromatography/Mass Spectrometry

The procedure was performed according to Foster et al., with minor modifications [52,53]. The monosaccharide fraction obtained from the hemicellulose hydrolysis (obtained in Section 3.3) was dissolved in deionized water (30 mL) and treated with NaBH_4_ (1 g, 26.4 mmol) before being stirred at r.t. for 2 h. Then, the reaction was quenched by the careful addition of glacial acetic acid (10 mL) while keeping the temperature under 30 °C by using external cooling. The solvent (water) and the excess of acetic acid were removed by evaporation under reduced pressure, and the obtained powder was treated with pyridine (30 mL) and acetic anhydride (30 mL). with stirring at a reflux for 1 h. Hence, the reaction was concentrated to dryness under reduced pressure, and the residue was partitioned between ethyl acetate (70 mL) and water (100 mL). The aqueous phase was extracted again with ethyl acetate (50 mL) and the combined organic phases were washed in turn with saturated NaHCO_3_ aq. (100 mL) and with saturated NaCl aq. (100 mL). The resulting solution was dried (Na_2_SO_4_) and concentrated in vacuo. The residue contained the alditol acetates of the hemicellulose monosaccharides, whose relative composition was determined by GC-MS analysis. GC-MS analyses for the determination of sugar composition in hemicellulose were performed on an HP-6890 gas chromatograph equipped with a 5973 mass detector and using an HP-5MS column (30 m × 0.25 mm, 0.25 µm film thickness; Hewlett Packard, Palo Alto, CA, USA). The temperature program was as follows: 120 °C (3 min)—12 °C/min—195 °C (10 min)—12 °C/min—300 °C (10 min); carrier gas: He; constant flow: 1 mL/min; and split ratio: 1:30. The reference standards of the alditol acetates were prepared, starting from the corresponding monosaccharides following the reduction/acetylation protocol described above. Compound identification was preliminary performed by comparing the MS data with the National Institute of Standards and Technology (NIST) database, and selected peaks were then confirmed with known standards (comparing both mass spectrum and retention times). The retention times of alditol acetates monosaccharides are given as follows: rhamnose—11.0 min; arabinose—11.2 min; xylose—11.5 min; mannose—15.7 min; glucose—15.8 min; and galactose—16.2 min.

### 3.6. Lignin Characterization

#### 3.6.1. Folin–Ciocalteu Analysis

The total phenolic content of the lignins was determined via a modified Folin–Ciocalteu (FC) protocol with some adaptation to the sample preparation step, as previously described [54]. Briefly, the samples were dissolved in DMSO with a final concentration of 2 mg/mL. For each determination, 5 μL of working solution (or the standard solution) was mixed with 120 μL of deionized water and 125 μL of FC reagent (Sigma 47641, Darmstadt, Germany) and then kept for 6 min at room temperature after 30 s of vortex stirring. Then, after the addition of 1.25 mL of 5% sodium carbonate and mixing, the vial was incubated in a thermoshaker at 40 °C for 30 min. The reaction mixture absorbance was measured using a UV–Vis spectrophotometer (Jasco V-560, Easton, PA, USA) equipped with a temperature-controlled cuvette holder and a thermostatic water bath (Haake K10, Karlsruhe, Germany). All the spectrophotometric measurements were carried out at 760 nm and 25 °C using a 1 cm optical path cuvette and deionized water as the blank sample. Vanillin was chosen as the reference standard. The calibration curve was constructed with nine different vanillin solutions in DMSO with a concentration in the range 0–500 μg/mL (see Supplementary Materials Figure S2). Each FC assay determination was carried out in triplicate.

#### 3.6.2. Molar Mass Determination

Gel permeation chromatography (GPC) was used to estimate the number- and weight-average molecular weights (M_n_ and M_w_, respectively) of the extracted lignins. GPC chromatograms were collected as 200 μL samples (concentration: 2 mg/mL) by means of a 510 HPLC system. The analyses were performed at 35 °C at a flow rate of 1 mL/min using tetrahydrofuran (THF) as the eluent. Prior to analysis, all samples were acetylated by following a procedure described in previous works [55].

#### 3.6.3. Fourier Transform Infrared Spectroscopy Analysis

Fourier transform infrared (FTIR) spectroscopy was used to assess the chemical composition of the extracted lignin samples. IR spectra were collected by means of a Nicolet iS50 FTIR spectrometer (Thermo Fisher, Waltham, MA, USA). The analyses were performed in air at room temperature by recording 64 scans at a resolution of 4 cm^−1^ in the 4000–600 cm^−1^ wavenumber range. All spectra were normalized with respect to the signal at 1515 cm^−1^, which is related to lignin aromatic ring vibrations and taken as the invariant band.

#### 3.6.4. Differential Scanning Calorimetry Analysis

Differential scanning calorimetry (DSC) analysis was used to determine the glass transition temperature of the extracted lignins. DSC curves were collected on ~15 mg samples by means of a DSC823e differential scanning calorimeter (Mettler-Toledo, Schwerzenbach, Switzerland). The analyses were performed under a N_2_ flux at a scan rate of 20 °C/min, using the following run: 25 °C → 180 °C → 25 °C → 250 °C. The glass transition temperature T_g_ was determined as the inflection point of the DSC trace recorded in the second heating run.

#### 3.6.5. ^13^C CP-MAS NMR Analysis

Solid-state magic-angle spinning (MAS) NMR experiments were carried out on a BRUKER NEO spectrometer (Bruker, Billerica, MA, USA) equipped with a commercial 4 mm MAS iProbe. The magnetic field strength was 11.74 T corresponding to a ^13^C NMR resonance frequency of 125.75 MHz. The solid sample was packed into a 4 mm ZrO_2_ rotor and spun at the magic angle with a spinning speed of 8 kHz at room temperature. The ^13^C CP-MAS spectra were acquired with a contact time of 1.5 ms, a repetition time of 4 s, and 17,000 scans. During acquisition, a proton two-phase pulse-modulated (TPPM) decoupling sequence was used [56].

## 4. Conclusions

This study addressed the efficient fractionation of five major biomasses derived from the agri-food industry, employing deep eutectic solvents (DESs). A comparative analysis was performed on DES-assisted fractionation using either conventional batch heating or microwave-assisted technology. The latter approach aimed to enhance process performance and sustainability, providing a promising basis for future industrial-scale applications and scale-up of this methodology. All recovered fractions from both fractionation strategies were comprehensively characterized. Notably, ^13^C CP-MAS NMR analysis of the recovered lignins provided a distinctive fingerprint of the biomass source. The synergistic exploitation of the microwave/DES-assisted process offered significant advantages over conventional heating, including faster heating rates, shorter reaction times, improved energy efficiency, and more effective disruption of the biomass matrix, collectively resulting in higher extraction yields. The outcomes in terms of final yields achieved through the second process were markedly improved compared to the first approach, thereby supporting its potential scalability in line with green chemistry principles. Complete fractionation of the biomasses was achieved with no residual material. Furthermore, the exploitation of the polysaccharide fractions in the production of recycled paper sheets has been demonstrated at laboratory scale, while the lignin fractions, with their content of functional groups, will be explored for the development of innovative bio-based polymeric materials, highlighting the potential of this integrated strategy within a zero-waste, circular bioeconomy framework.

## Figures and Tables

**Table 1:** Composition of the 5 studied biomasses (% *w*/*w*). Rapeseed water-soluble fraction (*) is composed of 36% proteins and 12% soluble sugars.

Sample	Water-Soluble Fraction	Hemicellulose	Cellulose	Lignin	Inorganic Residue
BSG	25	28	25	22	≤1
r-Rice Husk	6.5	19.5	43	19	12
p-Rice Husk	6.5	27	36	18.5	12
Rapeseed	48 *	10	10	15	2
Hemp	13	25	27	26	9

**Table 2:** Relative abundance of the monosaccharides deriving from hemicellulose hydrolysis.

Sample	Rhamnose	Fucose	Arabinose	Xylose	Mannose	Glucose	Galactose
BSG	3.0	1.0	29	65	-	-	2.0
r-RH	0.4	0.5	49.0	44.9	-	1.2	4.0
p-RH	0.2	0.5	22.6	69.2	-	4.9	2.6
Rapeseed	-	-	47	27	8.0	1.0	17.0
Hemp	-	-	34	60	2.0	1.0	3

**Table 3:** Composition of polysaccharide-enriched fractions from the different biomasses obtained using CHM and MWT technology. * Reported ratios of hemicellulose (H) and cellulose (C).

Biomass	Heating Mode	Arabinose	Xylose	Galactose	Mannose	Glucose	Ratio H/C *	Ratio C/H *
BSG	CHM	13	11	0	0	76	0.32	3.13
	MWT	15	43	0	0	42	1.38	0.72
r-RH	CHM	15	14	0	0	71	0.48	2.08
	MWT	16	21	0	0	63	0.59	1.70
p-RH	CHM	6	13	0	0	81	0.24	4.17
	MWT	8	40	0	0	52	0.92	1.10
Rapeseed	CHM	31	19	12	15	23	3.41	0.29
	MWT	43	25	11	2	19	4.39	0.23
Hemp	CHM	11	24	0	0	65	0.54	1.85
	MWT	16	32	0	0	52	0.90	1.11

**Table 4:** Number average molecular weight (Mn), weight average molecular weight (Mw), and polydispersity index (Ð) of all examined lignins. All samples were eluted after acetylation; reported values are relative to polystyrene standards. CHM samples were obtained by the conventional heating method; MWT samples were obtained by microwave-mediated treatment.

Lignin Sample		M_n_ (g/mol)	M_w_ (g/mol)	*Ð*
*Source*	*Heating Mode*			
BSG	CHM	820	1580	1.93
	MWT	1080	1560	1.36
r-RH	CHM	1360	5195	3.82
	MWT	1590	6260	3.94
p-RH	CHM	1310	3860	2.95
	MWT	1235	3790	3.07
Rapeseed	CHM	840	1710	2.04
	MWT	810	1660	2.05
Hemp	CHM	1315	3090	2.35
	MWT	1365	3210	2.35
Protobind 1000		830	2800	3.37

**Table 5:** Results of the determination of phenolic hydroxyl groups expressed as vanillin equivalents/g of lignin samples. CHM samples were obtained by the conventional heating method; MWT samples were obtained by microwave-mediated treatment. Estimated standard errors ± 0.1 mmol/g vanillin equivalent (1σ, from calibration data).

Lignin Sample		Vanillin Equivalent Content (mmol/g)
*Source*	*Heating Mode*	
BSG	CHM	1.2
	MWT	1.4
r-RH	CHM	1.6
	MWT	1.9
p-RH	CHM	1.6
	MWT	2.3
Rapeseed	CHM	2.8
	MWT	3.8
Hemp	CHM	1.7
	MWT	2.2
Protobind 1000		3.1

**Table 6:** Signal assignments for FTIR spectra (reported in Figure 6) of lignin extracted from different lignocellulosic biomasses.

Signal (cm^−1^)	Assignment
3400	O–H stretching
2925	C–H stretching of methyl and methylene groups
2850	
1744	C=O stretching of nonconjugated (1744 and 1712 cm^−1^) and conjugated (1656 cm^−1^) carbonyl/carboxyl groups in carbohydrate species
1712	
1656	
1600	Aromatic skeletal vibrations
1515 (reference)	
1460	C–H bending of methyl and methylene groups
1424	Aromatic skeletal vibrations (ring stretching coupled to C–H bending)
1380	Ring breathing of syringyl units with C–O stretching
1266	Ring breathing of guaiacyl units with C–O stretching
1214	C–C and C–O stretching of guaiacyl and condensed guaiacyl units
1125	C–H bending of syringyl units
1090 (shoulder)	C–O stretching of secondary alcohols and aliphatic ether
1033	C–H bending of guaiacyl units with C–O stretching of primary alcohols
833	C–H bending of guaiacyl and syringyl units

**Table 7:** Glass transition temperature (T_g_) values for all the extracted lignin samples. CHM samples were obtained by the conventional heating method; MWT samples were obtained by microwave-mediated treatment.

Lignin Sample		T_g_ (°C)
*Source*	*Heating Mode*	
BSG	CHM	151
	MWT	139
r-Rice Husk	CHM	161
	MWT	151
p-Rice Husk	CHM	162
	MWT	146
Rapeseed	CHM	104
	MWT	102
Hemp	CHM	150
	MWT	150
Protobind 1000		151

## Data Availability

The original contributions presented in this study are included in the article/supplementary material. Further inquiries can be directed to the corresponding authors.

## Supplementary Materials

The following supporting information can be downloaded at: https://www.mdpi.com/article/10.3390/molecules30173588/s1, Figure S1: ^1^H NMR spectrum of DES choline chloride/l-lactic acid (1:5 mol/mol); Figure S2: Calibration curve of Folin–Ciocalteu phenol titration with vanillin; Figure S3: GPC chromatogram graphs of lignin extracted from different lignocellulosic biomasses; Figure S4: ^13^C CP-MAS NMR spectra of lignins from (A) r-Rice Husk and (B) p-Rice Husk subjected to two different treatments (conventional heating and microwave heating); Figure S5: Comparison between ^1^H NMR spectra of DES choline chloride/l-lactic acid (1:5 mol/mol) before (blue spectrum) and after biomass fractionation (red spectrum); Figure S6. Process condition optimization performed on rapeseed as the reference biomass; Figure S7. Plot of T_g_ values vs. 1/M_n_ for the CHM (graph on the left) and MW (graph on the right) methods.
