# Supplementary material for: Synergistic DES–Microwave Fractionation of Agri-Food Biomasses in a Zero-Waste Perspective
Source: Molecules. 2025 Sep 2;30(17):3588. doi: 10.3390/molecules30173588 (PMC12430255; doi:10.3390/molecules30173588)
Supplement: Supplementary file 1 [file molecules-30-03588-s001.zip › molecules-3790211-supplementary .pdf]

Supplementary Materials

Figure S1. ^{1}H NMR spectrum of DES choline chloride/L-lactic acid (1:5 mol/mol). 2

Figure S2. Calibration curve of Folin-Ciocalteu phenol titration with vanillin. 3

Figure S3. GPC Chromatogram graphs of lignin extracted from different lignocellulosic biomasses. 4

Figure S4. ^{13}C CP-MAS NMR spectra of Lignins from A) r-Rice Husk and B) p-Rice Husk subjected to two different treatments (conventional heating mode CHM and microwave treatment MWT). 5

Figure S5. Comparison between ^{1}H NMR spectrum of DES choline chloride/L-lactic acid (1:5 mol/mol) before (blue spectrum) and after the biomass fractionation (red spectrum). 6

Figure S6. Process condition optimization performed on Rapeseed as reference biomass. 7

Figure S7. Plot of T_g values *vs* $1/M_n$ for CHM (graph on the left) and MWT (graph on the right) methods. 8

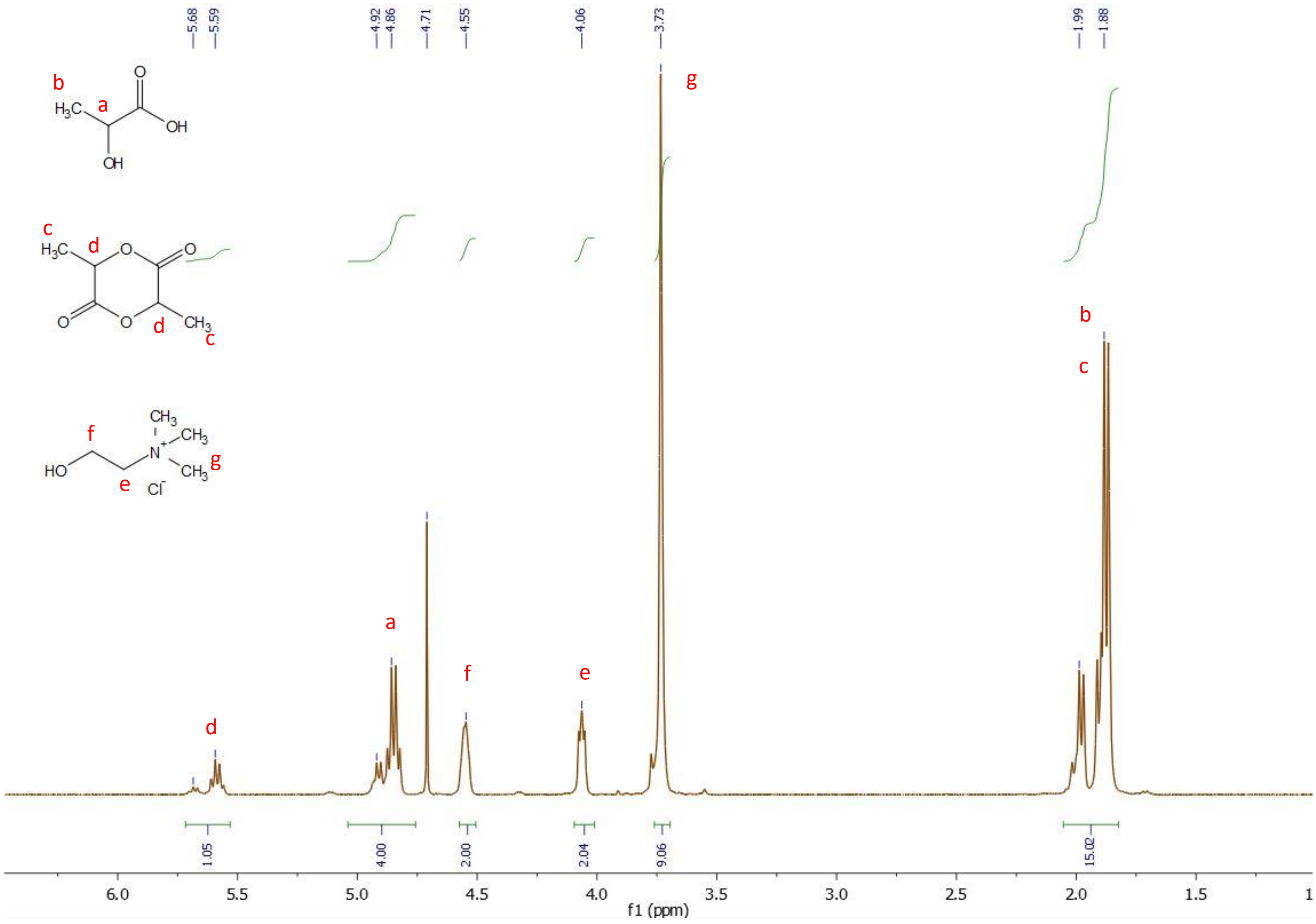

Figure S1. a) ^{1}H NMR spectrum of DES choline chloride/L-lactic acid (1:5 mol/mol).

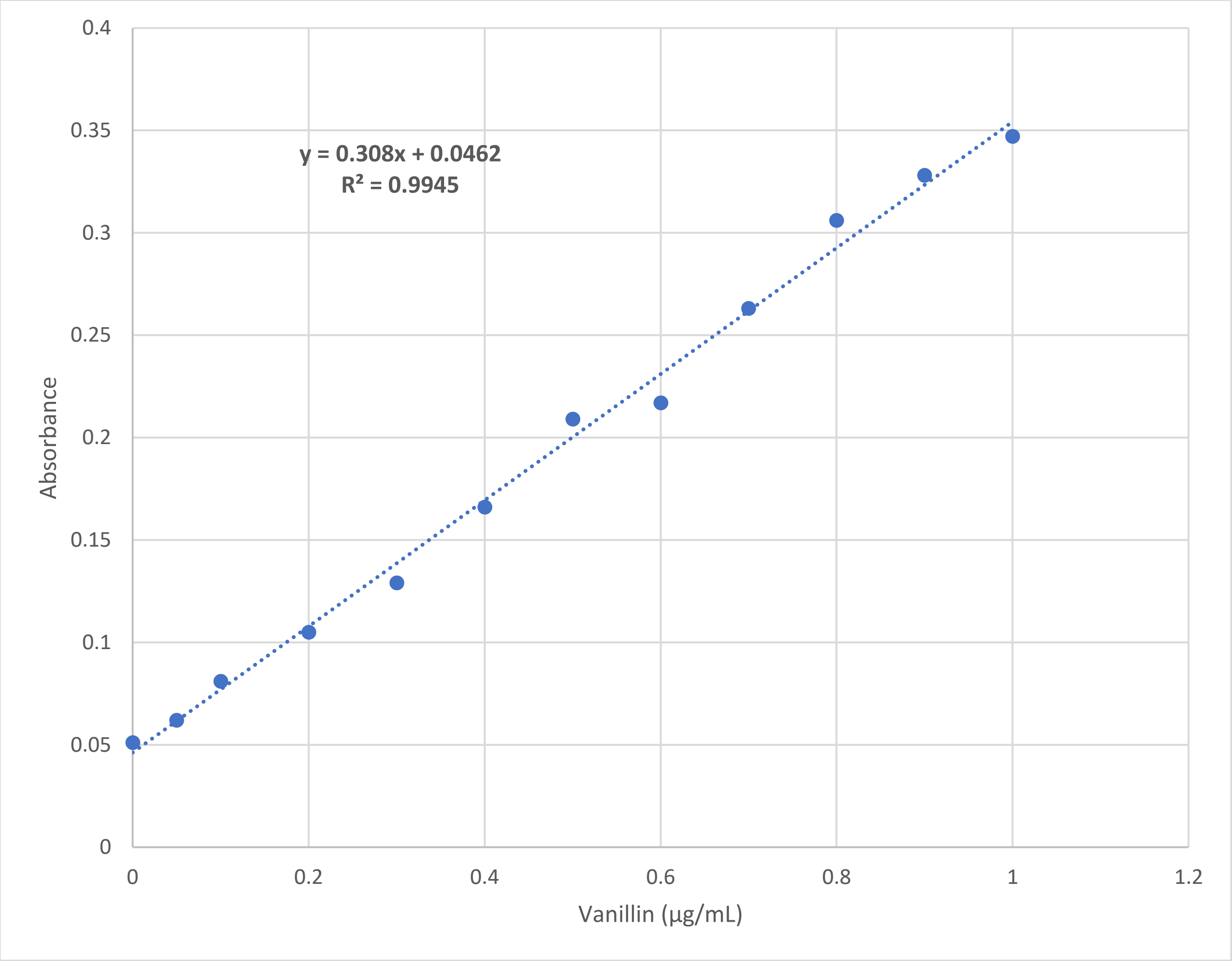

Figure S2. Calibration curve of Folin-Ciocalteu phenol titration with vanillin.

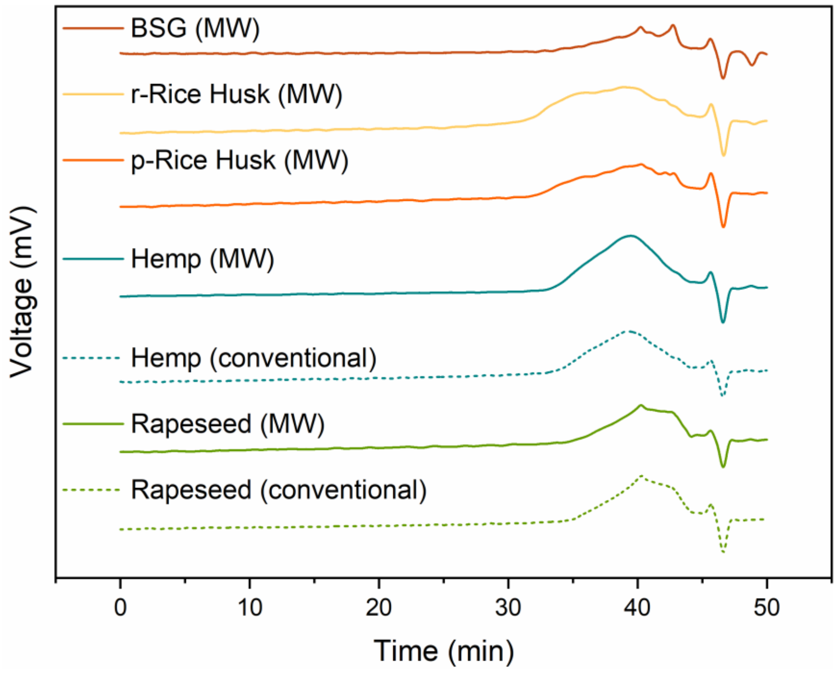

Figure S3. GPC Chromatogram graphs of lignin extracted from different lignocellulosic biomasses.

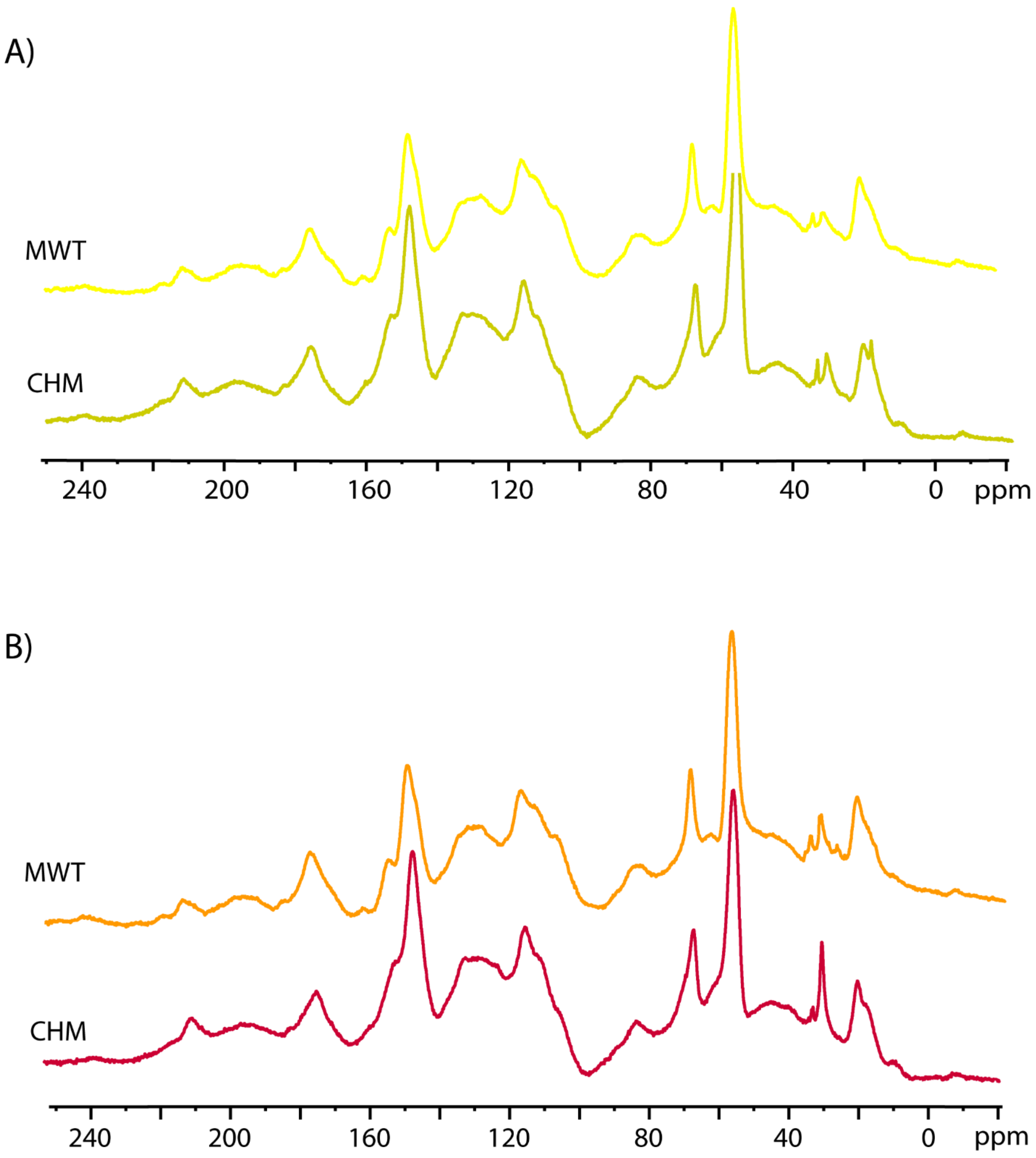

Figure S4. ^{13}C CP-MAS NMR spectra of Lignins from A) r-Rice Husk and B) p-Rice Husk subjected to two different treatments (conventional heating CHM and microwave heating MWT).

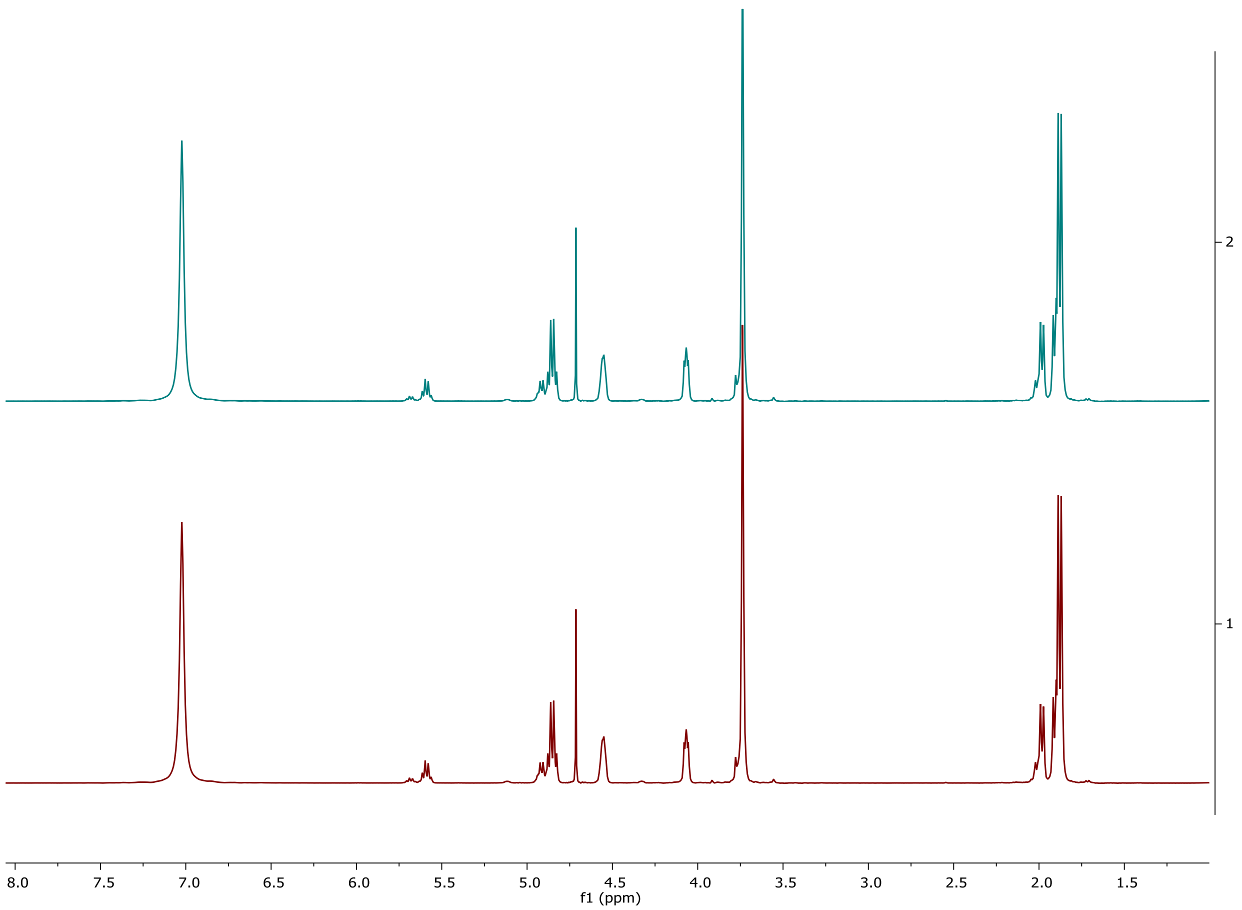

Figure S5. Comparison between ^{1}H NMR spectrum of DES choline chloride/L-lactic acid (1:5 mol/mol) before (blue spectrum) and after the biomass fractionation (red spectrum).

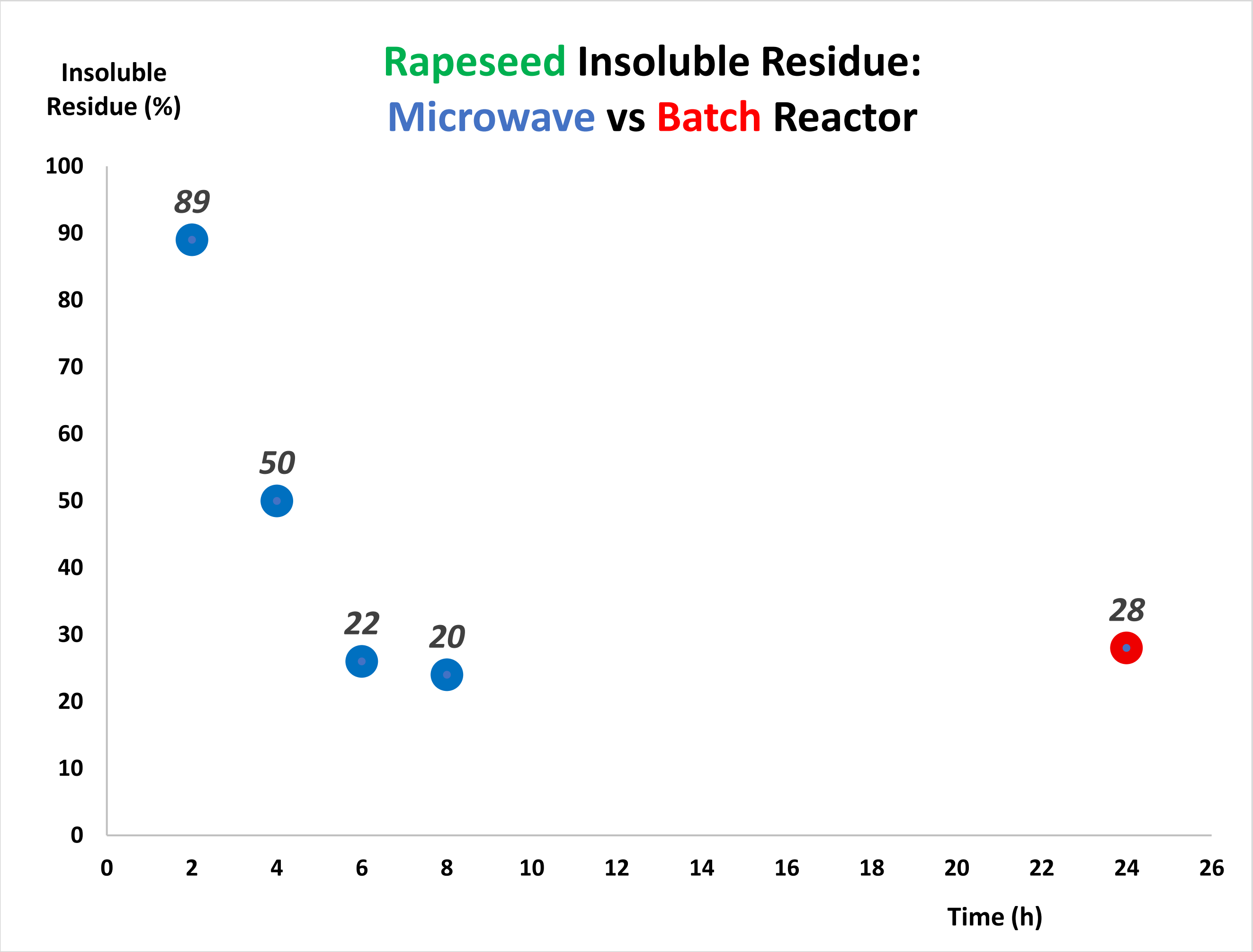

Figure S6. Process condition optimization performed on Rapeseed as reference biomass (optimization of the residence time for MWT process (blue points). It has been developed analyzing the insoluble residues of biomass during the time, keeping the set-point parameter the obtained insoluble residue observed from the standard 24 h CHM fractionation (red point)).

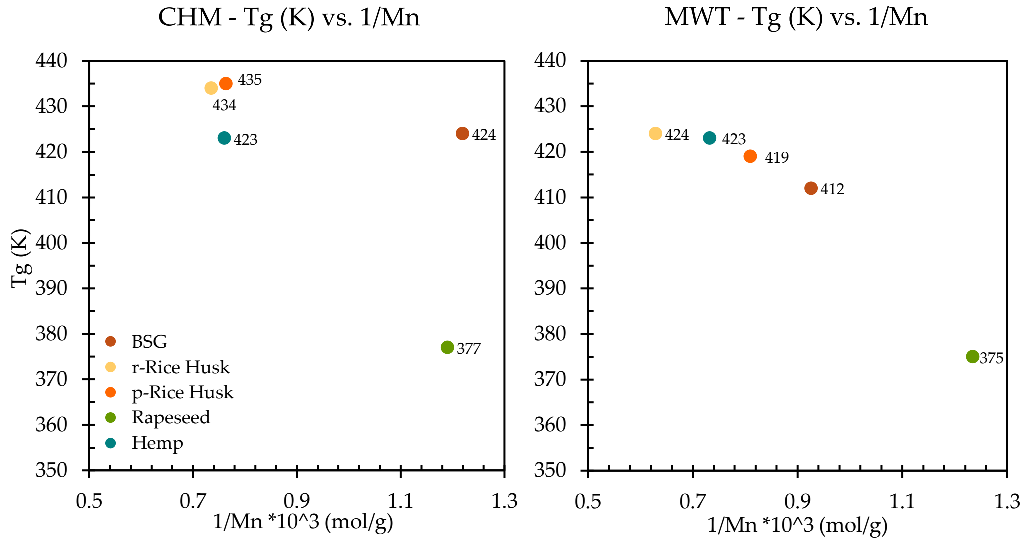

Figure S7. Plot of T_g values *vs* $1/M_n$ for CHM (graph on the left) and MWT (graph on the right) methods.
